# Screening of SERT and p11 mRNA Levels in Airline Pilots: A Translational Approach

**DOI:** 10.3389/fpsyt.2022.859768

**Published:** 2022-03-23

**Authors:** Enrique Becerril-Villanueva, María Irma Olvera-Alvarez, Samantha Alvarez-Herrera, Jose Luis Maldonado-García, Adolfo López-Torres, Oscar Abelardo Ramírez-Marroquín, Octavio González-Ruiz, José Manuel Nogueira-Fernández, José Manuel Mendoza-Contreras, Héctor Omar Sánchez-García, José Antonio José-Alfallo, Atenodoro Valencia Baños, Ana Berta Torres-Serrano, Janeth Jiménez-Genchi, Danelia Mendieta-Cabrera, Gilberto Pérez-Sánchez, Lenin Pavón

**Affiliations:** ^1^Laboratorio de Psicoinmunología, Instituto Nacional de Psiquiatría Ramón de la Fuente Muñiz, Ciudad de México, Mexico; ^2^Dirección General de Protección y Medicina Preventiva en el Transporte, Secretaría de Comunicaciones y Transportes, Ciudad de México, Mexico; ^3^Centro de Investigaciones Científicas, Instituto de Química Aplicada, Universidad del Papaloapan, Oaxaca, Mexico; ^4^Hospital Psiquiátrico Fray Bernardino Álvarez, Ciudad de México, Mexico; ^5^Servicios Clínicos, Instituto Nacional de Psiquiatría Ramón de la Fuente Muñiz, Ciudad de México, Mexico

**Keywords:** stress, depression, airline pilots, serotonin transporter, p11

## Abstract

Airline pilots are frequently exposed to numerous flights per week, changes in their circadian rhythms, and extended periods away from home. All these stressors make pilots susceptible to developing psychiatric disorders. Recently, emphasis has been placed on the need for molecular tests that help in the diagnosis of depression. The genes *SLC6A4* and *S100A10* encode serotonin transporter (SERT) and p11 protein, respectively. Their expression has been frequently associated with stress and depression. In this work, we quantified, by quantitative PCR, the expression of SERT and p11 in peripheral mononuclear cells of airline pilots compared to patients with depression and healthy volunteers. Moreover, by mass spectrometry, we quantified the serum serotonin levels in the same three groups. We found that SERT and p11 were overexpressed in the mononuclear cells of airline pilots and depressed patients compared to healthy volunteers. Although serum serotonin was not different between healthy volunteers and airline pilots, a decreasing trend was observed in the latter. As expected, serum serotonin in the patients was significantly lower. Alterations in SERT and p11 in airline pilots could be related to professional stress, a condition that could potentially affect their long-term mental health.

## Introduction

Depression is a mood disorder characterized by the presence of symptoms such as deep sadness, loss of interest, difficulty experiencing pleasure, and feelings of guilt or low self-esteem. Other sleep and appetite disorders as well as feelings of fatigue and changes in body weight may also occur. Depression has a high prevalence, is the leading cause of disability, and contributes significantly to the global burden of disease. Additionally, it affects people of all ages (1–3) and the most serious cases can result in suicide. The etiology of depression is complex, involving several genetic, biological, and psychosocial factors. Among the biological factors are monoamine deficiency, neurotrophic disturbances, dysfunctional HPA activity, and inflammatory alterations. Since the etiology of depression is multifactorial, much remains to be studied about the molecular mechanisms underlying the pathophysiology of this disorder (4, 5).

In recent years, airplane accidents have raised concerns about aviation security and the mental health of airline pilots. In 2015, Flight 9,525 crashed in a mountainous region of France through a suicidal act of the pilot, who was suffering from depression (6). As a consequence, the European Aviation Safety Agency (EASA) recommended increasing aviation safety and improving the diagnostic methods for psychiatric disorders (7). Airline pilots live with high levels of stress due to their occupation (8); however, other factors, such as their social life and family problems, also contribute to their stressful life (9, 10). Several authors have stated that all of these factors make pilots susceptible to developing psychiatric disorders such as depression (10). The prevalence of depression in airline pilots is 1.9–12.6% (11) compared to 7.2–12.9% reported in the general population (12). The discrepancy in the prevalence of depression between airline pilots and the general population suggests that this disorder could be underdiagnosed in pilots. Currently, the diagnosis of depression is performed by a psychiatrist, according to the Diagnostic and Statistical Manual 5th edition (DSM-5), using structured interviews and rating scales, such as Mini-Mental State Examination (MMSE), Beck depression inventory (BDI), and Hamilton Depression Rating Scale (HDRS). In the past 50 years, several research projects have focused their efforts on searching for potential biomarkers of depression (13), such as serum biomarkers (14), catecholamine metabolites (15), among others (1, 2). From the development of the real-time quantitative PCR analysis (RT-qPCR), quantitative gene expression analysis has become an important tool in the search for biomarkers (14, 16–18). Both serotonin transporter (SERT) and p11 are proteins closely related to stress and depression (19–21). In lymphocytes of depressed patients, the increased gene expression of SERT (16, 18, 22) has been reported; meanwhile, p11 has been identified as a regulator of depressive-like behavior in animal models, showing a depressant (23) or anti-depressant (24) activity, depending on the brain region in which it is expressed. Moreover, the brain regions of subjects who have attempted suicide have shown reductions in p11 mRNA levels (24), and the protein has been proposed as a biomarker of post-traumatic stress disorder (PTSD) (20).

In this brief research report, we analyzed the gene expression of SERT and p11 in peripheral blood mononuclear cells (PBMCs) and measured the serum levels of serotonin in airline pilots compared to depressed patients and healthy volunteers. We found an increase in the gene expression of SERT and p11 in the PBMCs of airline pilots, as well as a decreasing trend in their serum serotonin levels.

## Materials and Methods

### Participant Recruitment

**Figure 1:**
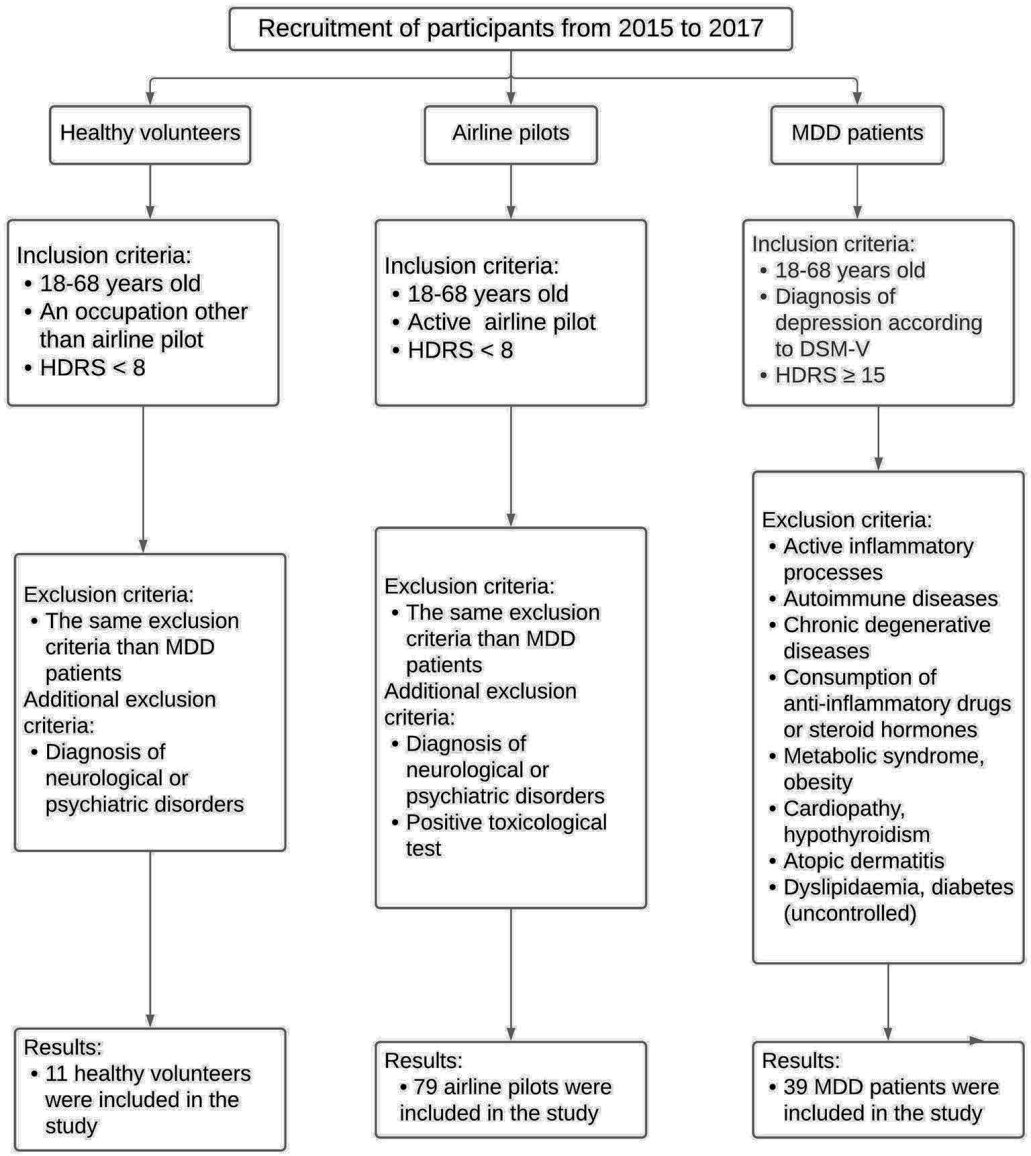
Flow diagram of participant recruitment. The figure describes the inclusion and exclusion criteria for healthy volunteers, airline pilots, and MDD patients.

Airline pilots (AP), healthy volunteers (HV), and patients with depression (MDD) were recruited from 2015 to 2017 according to the protocol NC16044.0. All the participants agreed to participate in this study and signed the informed consent forms. They were evaluated by a certified psychiatrist applying the MMSE (Mini-Mental State Examination), BDI (Beck Depression Inventory), and HDRS (Hamilton Depression Rating Scale). The protocol NC16044.0 was approved by the corresponding research and ethics committees of the Instituto Nacional de Psiquiatría Ramón de la Fuente Muñíz (INPRFM), according to the international guidelines. The AP were recruited at the Secretaría de Comunicaciones y Transportes (SCT), Mexico, while HV and MDD were recruited at the INPRFM and Hospital Psiquiátrico Fray Bernardino Álvarez, Mexico. The inclusion and exclusion criteria are described in the flow diagram of Figure 1.

### PBMCs and RNA Isolation

We collected peripheral blood samples (10 mL) in tubes with sodium heparin (Becton Dickinson Vacutainer®, USA) by venepuncture. We isolated PBMCs using Ficoll-Histopaque (Histopaque-1077, Sigma-Aldrich) according to the manufacturer's instructions. PBMC isolation was performed within 2 h after blood sample collection. Immediately, the PBMC-containing pellet was homogenized in 1 mL TRIzol® and RNA isolation was performed according to the manufacturer's instructions. Samples were then stored at −80°C until cDNA synthesis.

### Quantitative PCR of Serotonin Transporter and p11

The cDNA was synthesized from 1 μg total RNA pre-treated with 1 μL (1 U) DNase I (Invitrogen). Reverse transcription was performed using 1 μL (200 U) of MMLV reverse transcriptase (PROMEGA) according to the manufacturer's instructions. All qPCR reactions performed used 50 ng cDNA as template. The probes we used were Hs00169010_m1 (*SLC6A4: gene name of serotonin transporter*), Hs00751478_s1 (*S100A10: gene name of p11*), and Hs01060665_g1 (*ACTB: gene name of* β*-actin)*, as well as TaqMan® Master Mix (ThermoFisher®). All assays were made in duplicate using CFX96^TM^ REAL TIME SYSTEM® (BIORAD). We evaluated the system suitability for each qPCR assay by the *R*^2^ (≥0.99) and PCR efficiency (90–110%) parameters. The quantitative analysis was performed with the 2^−ΔΔCt^ method (25), using the ΔCt mean of healthy volunteers as a calibrator to calculate the ΔΔCt.

### Serum Serotonin Quantification by Liquid Chromatography Coupled to Mass Spectrometry

We measured serum levels of serotonin by reversed-phase Ultra High Performance Liquid Chromatography Electrospray-Ionization High Resolution Mass Spectrometry (UPLC-ESI-HRMS). Because not all serum samples were available, only 18 depressed patients, 14 airline pilots, and 7 healthy volunteers were included in this analysis. Firstly, we processed serum samples (1 mL) by adding 1 mL of a solution containing 5% citric acid, 2.5 mM L-lysine, and 2.5 mM EDTA. Then, proteins were precipitated with 200 μL 2.4 M perchloric acid at −20°C for 20 min. We obtained the supernatant containing serotonin by centrifugation at 14,000 × *g* at 4 °C for 15 min. After that, the serotonin was trapped and cleaned by solid phase extraction (SPE) using C_18_ cartridges (ThermoFisher®) and eluted with 250 μL acetonitrile. The serotonin extracts were vacuum dried and stored at −80°C until UPLC-ESI-HRMS analysis. Secondly, we resuspended serotonin extracts with 125 μL 0.1% (v/v) aqueous formic acid and filtered them through 0.22 μm PTFE membrane. We carried out the determination of serotonin (5-HT) by reversed-phase UPLC-ESI-HRMS according to the previous report (26) with modifications. In brief, we performed the analysis in an Acquity UPLC I-Class System (Waters) coupled to a Synapt G2-Si HDMS Q-TOF mass spectrometer (Waters) fitted with an ESI source. The LC-MS system and data were manipulated by MassLynx 4.1 software (Waters). The sample (5 μL) was injected onto a Luna Omega 1.6 μm C18 (2.1 × 150 mm, Phenomenex) using an isocratic separation with mobile phase composed of 4% (v/v) methanol in 0.1% aqueous formic acid delivered at a flow rate of 0.20 mL/min maintained at 40°C. The analysis time was 8 min and the column was cleaned between injections with 80% (v/v) methanol in 0.1% aqueous formic acid for 2 min. Ionization was performed in positive mode under the following conditions: capillary voltage, 3,000 V; capillary temperature, 120°C; sampling cone, 30 V; desolvation temperature, 300°C; and desolvation gas, 800 L/h. Full-scan mass spectra were acquired from 50 to 1,200 Da with acquisition data rate of 0.4 s and were corrected using leucine enkephalin as lock mass. For 5-HT quantification, the molecular ion [M+H-NH_3_]^+^ with exact mass 160.0762 was monitored.

### Statistical Analysis

We performed a normality test using the Kolmogorov-Smirnov test for all data. The results of psychiatric tests, qPCR, HPLC-mass spectrometry, and demographic data were analyzed by the non-parametric Kruskal-Wallis and Dunn's multiple comparison tests. Correlation analysis were done following Spearman's correlation. All the statistical analyses were performed using GraphPad Prism version 8.0.0 for Windows (GraphPad Software, San Diego, California USA, www.graphpad.com). Significance was stablished when *p* < 0.05.

## Results

### Participants and Demographic Data

We recruited a total of 146 airplane pilots from 2015 to 2017; however, 67 were excluded since they did not meet the inclusion criteria, and the final number of airline pilots was 79. Moreover, we recruited 11 healthy volunteers and 39 patients with diagnosis of major depressive disorder (MDD) that met the inclusion criteria (Figure 1). Table 1 shows the demographic data from healthy volunteers, airline pilots, and MDD patients.

**Table 1:** Demographic data and psychiatric tests of healthy volunteers, Airline pilots, and MDD patients.

**Group**	***n***	**Age: mean (SEM)**	**Gender: female/male**	**HDRS: mean (SEM)**	**BDI: mean (SEM)**
Healthy V (HV)	11	29.82 (1.39)	3/8	0.72 (0.42)	1.54 (0.83)
A. Pilots (AP)	79	37.32 (1.31)	2/77	1.92 (0.10)	0.87 (0.17)
Patients (MDD)	39	35.08 (1.75)	31/8	23.87 (1.06)^***^	29.97 (1.50)^***^

*** *p < 0.001: MDD vs. HV and MDD vs. AP*.

### Psychiatric Tests

No airline pilot or healthy volunteer was diagnosed with depression, or any other psychiatric disorder, according to the results of MMSE, BDI, and HDRS tests. As expected, depressed patients had significantly higher scores in BDI (*p* < 0.001) and HDRS (*p* < 0.001) as compared to airline pilots and healthy volunteers (Table 1). We found no significant correlation (*R* ≥ 0.8) between psychiatric scores of HDRS and BDI and the 2^−ΔΔCt^ values of p11 or SERT.

### Elevated mRNA Levels of SERT and p11 in PBMCs of Airline Pilots and Depressed Patients

**Figure 2:**
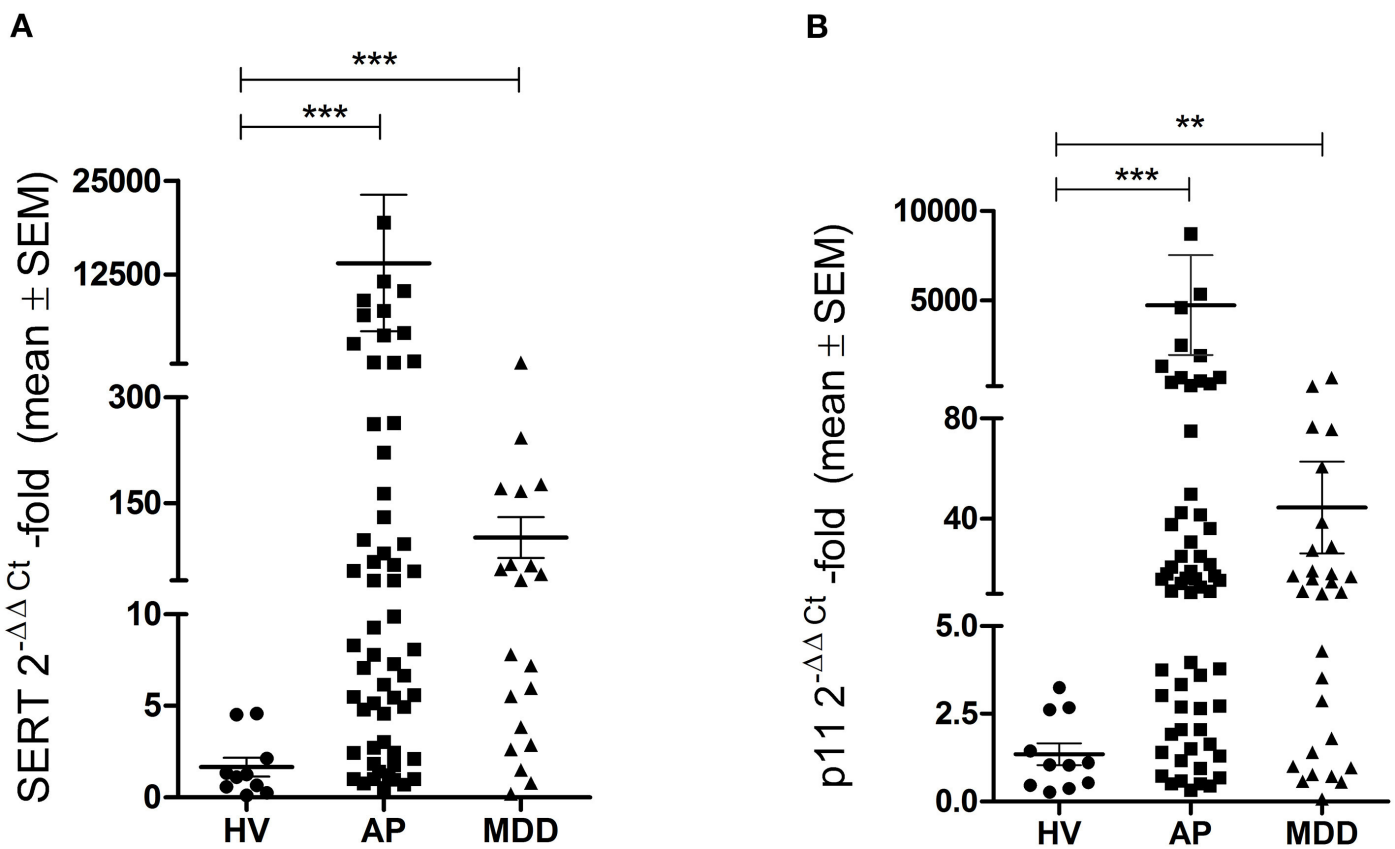
Gene expression of serotonin transporter and p11. Quantitative real time-PCR analysis of serotonin transporter **(A)** and p11 **(B)** in healthy volunteers (HV), airline pilots (AP), and depressed patients (MDD). Relative quantification performed by 2^−ΔΔCt^ method. Statistical analysis was performed by Kruskal-Wallis with Dunn's multiple comparison test (**p < 0.01; ***p < 0.001). SEM, standard error of mean.

The RT-qPCR analysis showed that the expression of SERT was significantly higher in depressed patients (*p* < 0.001) and airline pilots (*p* < 0.001) as compared to healthy volunteers, with mean (standard error of mean) 2^−ΔΔCt^ values of 101.3 (29.0), 13,986 (9,129), and 1.65(0.51), respectively (Figure 2A). The expression of p11 was also significantly higher in depressed patients (*p* < 0.01) and airline pilots (*p* < 0.001) compared to healthy volunteers, with mean 2^−ΔΔCt^ values of 44.52 (18.29), 4,740 (2,785), and 1.34 (0.31), respectively (Figure 2B).

### Serum Levels of Serotonin by UPLC-ESI-HRMS

**Figure 3:**
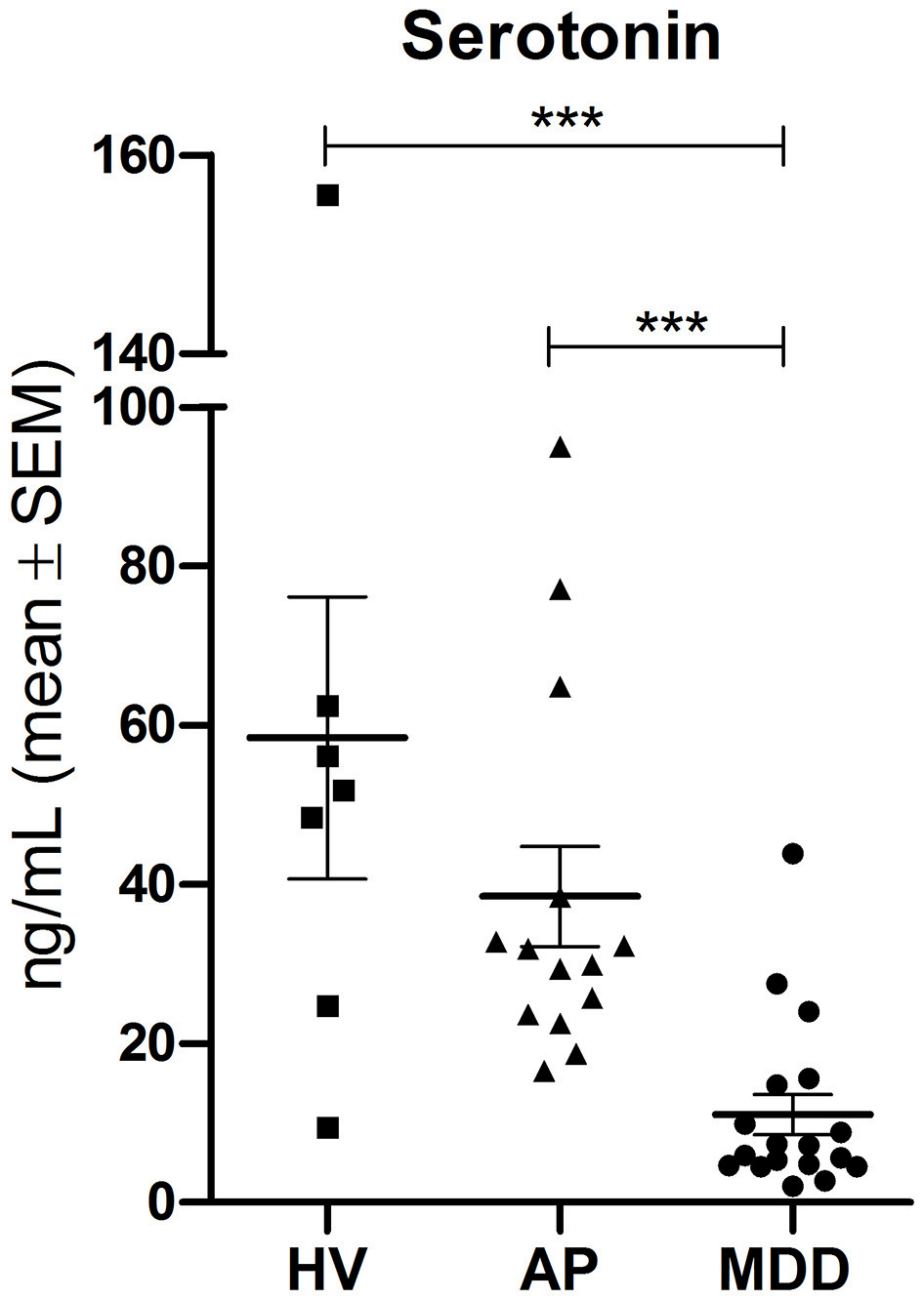
Serum serotonin levels. Serotonin quantification by UPLC-ESI-HRMS in healthy volunteers (HV), airline pilots (AP), and depressed patients (MDD). Statistical analysis was performed by Kruskal-Wallis with Dunn's multiple comparison test (***p < 0.001). SEM, standard error of mean.

We identified the full scan ESI-HRMS spectrum for serotonin, and it is shown in the Supplementary Figure 1. We found, as expected, that serum serotonin levels were significantly lower in depressed patients compared to airline pilots (*p* < 0.001) and healthy volunteers (*p* < 0.001), with mean values (standard error of mean) of 11.05 (2.54), 38.48 (6.27), and 58.42 ng/mL (17.75), respectively (Figure 3). Furthermore, we noted that the serum serotonin levels of airline pilots had a decreasing trend, although they were not statistically different or as low as those of depressed patients.

## Discussion

Since 1972, there have been 14 fatal events worldwide caused by the suicide of pilots while in flight, resulting in 562 deaths (27). Hence, airline pilots' mental health becomes particularly relevant in terms of aviation security. Factors such as the heavy workload and the frequent exposure to jet lag could contribute to a high level of stress and affect pilots' emotional stability. Stress and depression have such a close and strong relationship that the animal models used to study depressive disorders are based on stress models (28). Because of the stressful life of pilots (9), we decided to study their gene expression of SERT and p11 and their serum serotonin levels and compare them to those of depressed patients and healthy volunteers.

Our results showed that, as expected, HDRS and BDI scores were significantly higher in depressed patients than healthy volunteers and airline pilots. We must highlight that HDRS is a clinician-rated test, whereas BDI is a self-rated test. Thus, in HDRS, interpreting the psychiatrist is critical, while in BDI, the patient's perception of their symptoms is the most important. Moreover, many items of the HDRS scale focus on somatic symptoms of major depression, while BDI detects subjective/cognitive factors scarcely represented in HDRS (29, 30). The interpretive value required by clinical scales has motivated many authors to search for molecular parameters that may be useful in clinical diagnosis in conjunction with the psychiatric scales currently used.

In this sense, we found that airline pilots and depressed patients showed elevated mRNA levels of SERT and p11, compared to healthy volunteers. SERT and p11 have been strongly associated with stress and depression. Increased mRNA levels of SERT have been reported in lymphocytes of depressed patients (16, 18, 22); in contrast, other studies have reported reduced levels of the protein (21). Depressed patients have also been reported to show significantly reduced protein levels of SERT in brain regions such as the amygdala, striatum, and brainstem (31). Until now, the reason for the discrepancy between reduced protein levels and increased mRNA levels that have been reported in different works has not been fully clarified. For its part, p11 plays a role as a pharmacological mediator of antidepressants and regulator of serotonin receptor signaling and neurogenesis (19). Moreover, p11 has been proposed as a biomarker of post-traumatic stress (20). Recent studies using models of chronic unpredictable stress in mice have demonstrated the important role of p11 and epigenetic changes in its promoter in the development of depressive-like behavior (32). There is evidence that p11 interacts with 5-HT_1B_ and 5-HT_4_ serotonin receptors and modulates their function (33–35) besides inducing the expression of 5-HT_4_, but there is no evidence of the direct interaction between p11 and SERT.

To our knowledge, this is the first work to report high mRNA levels of SERT and p11 in PBMC from airline pilots. Both genes are related to depression (16, 19) and play key roles in the functioning and regulation of the serotonergic system (35, 36). A dysfunctional serotonergic system leads to abnormal neurotransmission and the development of psychiatric disorders. Although our study was performed on peripheral blood cells, some authors have proposed that alterations in PBMC could be a reflection of alterations in the central nervous system (CNS) (37–39).

On the other hand, although the serotonin levels in airline pilots were not statistically different from those in healthy volunteers, we want to highlight that its decreasing trend is concerning. Serotonin is an important neurotransmitter with many physiological effects, and its proper functioning is necessary for mental health (40–42). Interestingly, depressive and suicide attempt patients have been reported to show low serum serotonin levels (43). Postmortem studies have revealed that 20% of pilots who died in fatal accidents had psychopathology or used selective serotonin reuptake inhibitors (SSRIs), but this was not reported in their latest medical examinations (44, 45). However, it has been shown that pilots who spend many hours flying per week and are frequently exposed to jet lag or alterations in their circadian rhythm are more likely to experience depressive or anxious symptoms (46, 47). In fact, pilots themselves have associated their fatigue with sleep deprivation and a heavy workload (48). Airline pilots are likely to deny their depressive symptoms or the use of antidepressants for fear of being suspended from work, posing a great risk to aviation. This triggers alarms in terms of aviation security and points out the importance of paying greater attention to the health of the aviation guild and the improvement of their working conditions. In addition, some reports have highlighted the lack of social support as a factor contributing to the deterioration of the pilots' mental health (9, 11).

Although our groups are unbalanced and heterogeneous, the sample size effect in our study is small when comparing MDD vs. AP. There are more women in the MDD group, but they are a minority in airline pilots and healthy volunteers. This is mainly due to the nature of the groups: Depression is higher in females than males, while most airline pilots are male. We are aware further studies are required in a larger and more diverse cohort; however, we cannot change the gender proportions of the groups, since this is a natural phenomenon. The issue of the differential sensitivity between men and women to develop mood disorders has been addressed by Kendler et al. (49). Kendler et al. reported that males are prone to suffer from introjective depression and females from anaclitic depression. Men are more likely to be emotionally invested in job and financial success; thus, the stress due to occupational activities (49), as in the case of airline pilots, could seriously affect their mental health.

In conclusion, in this brief research report, we show that airline pilots and depressed patients have similar alterations in their SERT and p11 profiles, distinguishing them from healthy volunteers. These alterations in airline pilots could be related to professional stress. Notably, this is the first SERT and p11 screening in this guild, and this work's prospects are to explore their potential as biomarkers in a larger and more diverse cohort.

## Limitations of the Study

Future studies should consider the effect of sample size. Notably, there are more women in the group of patients with MDD while they are a minority in airline pilots and healthy volunteers. This is mainly due to the nature of the groups. We only use one housekeeping gene: β-actin. Due to their small size, platelets are a potential contaminant of PBMC. Still, the content of mRNA in platelets is considerably less than that of leukocytes; therefore, this greatly reduces contamination by genetic material.

## Data Availability Statement

The original contributions presented in the study are included in the article/Supplementary Materials, further inquiries can be directed to the corresponding authors.

## Ethics Statement

The studies involving human participants were reviewed and approved by Comité de Ética en Investigación del Instituto Nacional de Psiquiatría Ramón de la Fuente Muñiz, México. Protocol: NC16044.0; Ref. CEI/C/081/2015. The patients/participants provided their written informed consent to participate in this study.

## Author Contributions

LP, JJ-A, and GP-S: conceptualization. EB-V and MO-A: first draft of manuscript and experiments and data analysis. GP-S and EB-V: review and correction of manuscript. EB-V, MO-A, and SA-H: RT-qPCR experiments. SA-H and JLM: mononuclear cells and serum sample processing from patients and healthy volunteers. AL-T and OR-M: UHPLC-Mass spectrometry analysis. JJ-A: supervision and coordination of airline pilots' participation and depressed patients' participation. JM-C, OG-R, and JN-F: general administration and approval of the participation of airline pilots. MO-A and HS-G: mononuclear cells and serum sample processing of airline pilots. JJ-G, DM-C, AV-B, and AT-S: clinical evaluation of the participants. LP and GP-S: data curation and statistical analysis. LP: funding acquisition. All authors approved the final version of this manuscript.

## Funding

This research was funded by the Ramón de la Fuente Muñíz National Institute of Psychiatry; SECITI: NC150048, SECITI (SECITI 0048/2014), CONACyT, FOSISS: SALUD-2017-1-289800 (NC16044.0), and CONACyT # INFRA-2015-01-252013.

## Conflict of Interest

The authors declare that the research was conducted in the absence of any commercial or financial relationships that could be construed as a potential conflict of interest.

## Publisher's Note

All claims expressed in this article are solely those of the authors and do not necessarily represent those of their affiliated organizations, or those of the publisher, the editors and the reviewers. Any product that may be evaluated in this article, or claim that may be made by its manufacturer, is not guaranteed or endorsed by the publisher.

## Supplementary Material

Supplementary Figure 1 Full scan ESI-HRMS spectrum for serotonin. The most abundant peak (160.0762 m/z) corresponds to the fragmented ion of serotonin, whereas the least abundant (177.1025 m/z) corresponds to the precursor ion.
