# Supplementary figures and images for: Screening of SERT and p11 mRNA Levels in Airline Pilots: A Translational Approach
Source: Front Psychiatry. 2022 Mar 23;13:859768. doi: 10.3389/fpsyt.2022.859768 (PMC8983845; doi:10.3389/fpsyt.2022.859768)

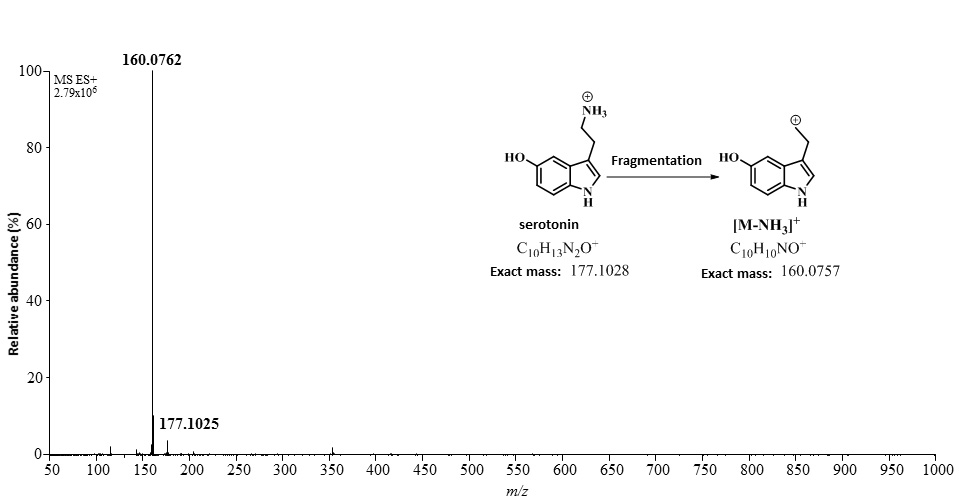

Supplement: Supplementary Figure 1 — Full scan ESI-HRMS spectrum for serotonin. The most abundant peak (160.0762 m/z) corresponds to the fragmented ion of serotonin, whereas the least abundant (177.1025 m/z) corresponds to the precursor ion. [file Image_1.JPEG]
